# Unravelling allergic rhinitis: exploring pathophysiology, advances in treatment, and future directions

**DOI:** 10.3389/falgy.2025.1636415

**Published:** 2025-12-04

**Authors:** Aryan Kumar Singh, Shradha Shaili, Ayesha Siddiqui, Ahsan Ali, Ananya Choubey, Pooja Jain, Mohd Aamir Mirza, Zeenat Iqbal

**Affiliations:** Jamia Hamdard University, New Delhi, India

**Keywords:** AR, allergen sensitization, respiratory infections, allergen immunotherapy, skin prick test

## Abstract

Allergic rhinitis (AR) is a complex, multifactorial condition that continues to pose significant clinical and public health challenges, despite the availability of established therapeutic strategies. It significantly contributes to a lower quality of life by causing sleep issues, mental fatigue, and a decline in productivity. A thorough grasp of AR is crucial to enhancing diagnosis and treatment results because of its pervasive effects and ongoing management gaps. This review covers a wide range of topics, such as classification schemes, historical perception, and physical consequences of AR. It talks about the etiological elements that influence the pathophysiology of the illness and sheds light on the immune systems at play. By critically examining current diagnostic limitations and barriers to early intervention, this review underscores the necessity for improved clinical awareness and patient education. Additionally, the paper assesses the variety of existing treatment options, ranging from allergy immunotherapy to pharmaceutical interventions, and investigates breakthroughs in the treatment of AR, including phytotherapy and innovative therapeutic techniques. Trends in patient preferences and clinical uptake are noted, along with the market's evolution for AR treatments. Furthermore, current clinical studies for possible pharmacotherapies are examined, highlighting the significance of continued innovation in the treatment of AR. The review's conclusion makes recommendations for enhancing clinical practice, public health initiatives, and patient outcomes as well as future research directions. By highlighting the necessity of improved clinical awareness and intervention techniques, this thorough analysis seeks to offer a comprehensive understanding of AR and its management.

## 1 Introduction

“Allergic Rhinitis is a chronic, IgE-mediated type 2 inflammatory disease that affects both adults and children. It imposes a significant financial burden on healthcare systems and negatively impacts patients’ quality of life” (1). An estimated 20%–40% of people globally have AR, the most common clinical symptom of respiratory allergies. In India, allergic rhinitis affects 20%–30% of people, and 15% of those people go on to develop asthma (2). The “one airway, one disease” approach demonstrates how AR is frequently linked to conjunctivitis and/or asthma (3).

The first year of life is when sensitization to inhaled allergens starts; indoor allergen sensitization comes before pollen sensitization (4). Identifying AR in the first two or three years of life is extremely challenging because viral respiratory infections are common in young children and elicit comparable symptoms. In the second to fourth decades of life, AR is most common, after which it gradually declines (5). The signs of rhinitis caused by AR commonly peak in the second, third, and fourth decades of life after beginning in early childhood. It is not unusual for this disease to start in childhood or later in adulthood, though. Although it has been shown that there are genetic determinants of total IgE levels and specific allergen sensitization, neither the kind of atopic illness nor specific sensitivities seems to be heritable as simple genetic traits. The chance of developing allergic AR was found to be correlated with polymorphisms of the transcription factors GATA binding protein 3, trans-acting T-cell-specific transcription factor, and interleukin (IL)-13 in a cohort of children followed up to age 10 (6).

Medical history, physical examination, and, if necessary, nasal endoscopy are used to diagnose AR. In certain cases, testing for allergen-specific IgE (skin prick or serum-specific IgE) is also performed. Table 1 depicts physical ruling and historical perceivance. Guidelines on AR and Its Impact on Asthma, which were released in 2001 in collaboration with the World Health Organization, recommend treating AR by combining immunotherapy, medication, allergen avoidance, and patient education. For instance, the absence of a definitive cure is often linked to poor adherence to long-term therapy, inadequate consideration of individual patient needs, and limited patient understanding of the disease (10, 11).

**Table 1:** AR: physical ruling and historical perceivance (7–9).

Outline of symptoms	Historical definitions	Physical ruling
Nasal symptoms Nasal congestion • Sneezing • Rhinorrhea • Itching of nose • Postnasal drip Non- nasal symptoms • Frequent throat clearing • Cough • Malaise • Fatigue • Eczema • Headache • Itching of ear and palate	• Seasonal vs perennial nature of symptoms • Manifestation on subjection to particular agent (animals, particular plants) • Prevalent therapy • Genealogy of atopic or allergic disease • Manifestation on irritant exposure • Manifestation of upper respiratory infection	• Clear rhinorrhoea • Pale or bruised nasal mucosal swelling • Ocular findings (watery discharge, swollen conjunctiva, scleral infection) • Frequent throat clearing • Nasal polyps • Allergic shiners • Absence of foreign body, tumour, purulence suggesting infection. • Fiberoptic visualization of nose • Lymphadenopathy suggest infectious cause of rhinitis

Despite the availability of therapeutic approaches for the treatment of AR, a substantial amount of the population is still uncontrolled. Further therapeutic advances and research are requisite for the treatment of AR. In this review, we have compiled the different aspects, including pathophysiology, diagnosis, commercially available formulations, and ongoing clinical trials for the treatment of AR.

## 2 Classification

**Figure 1:**
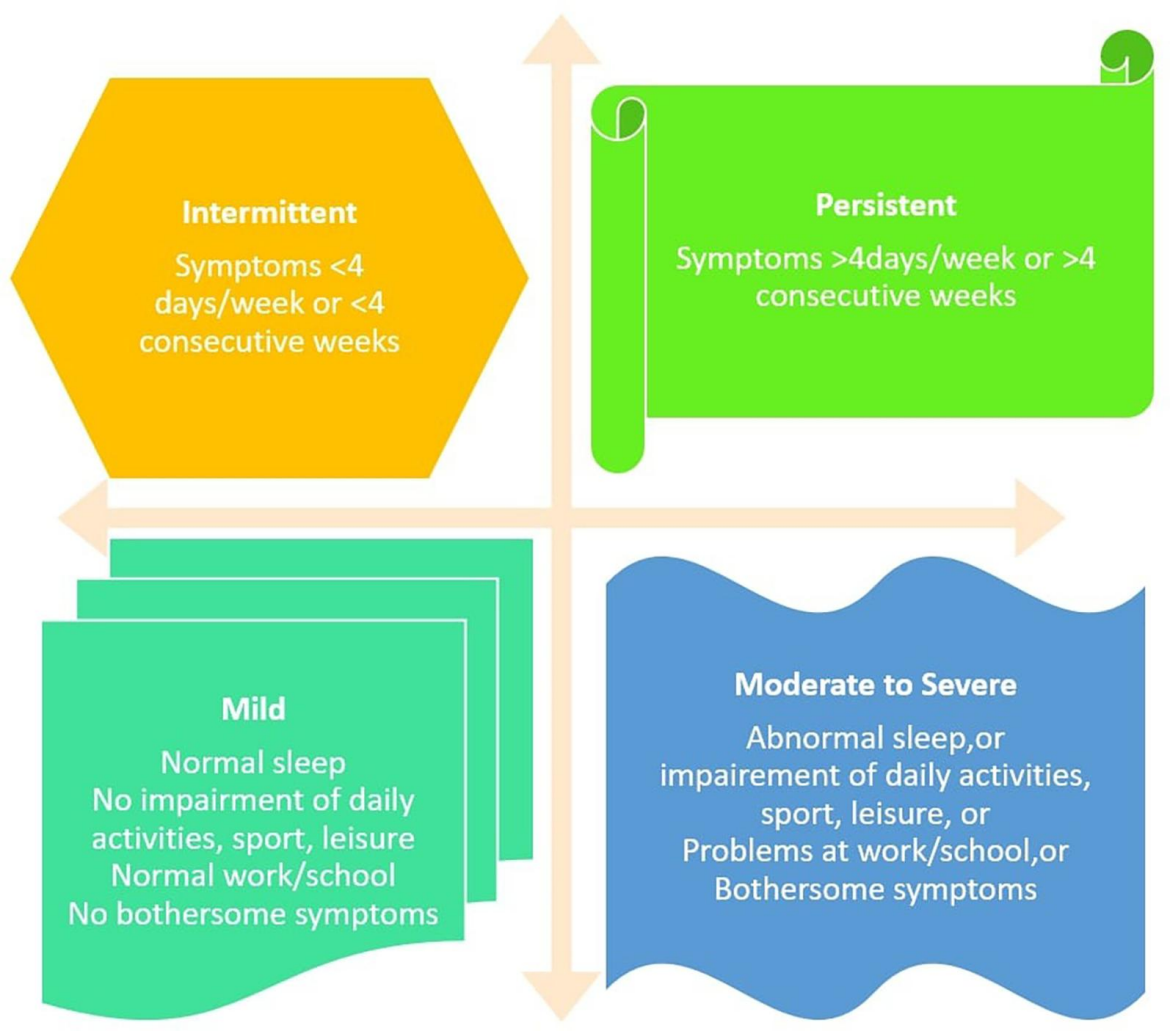
ARIA classification of AR (13).

AR has traditionally been classified as seasonal or perennial based on an individual's sensitivity to either cyclic pollens or year-round allergens such as dust mites, animal dander, cockroaches, and moulds (12). Following the internationally prescribed ARIA (AR and its impact on asthma) guidelines for the classification and management of AR, allergic nasal illness is now classified as mild or moderate-to-severe, intermittent or persistent. Figure 1, illustrated below, depicts the ARIA classification of AR. Pharmacotherapy can be established with the use of this classification (14). Table 2 depicts the etiological classification of AR.

**Table 2:** Etiological classification of rhinitis (15).

S. No	Types of AR	Criteria of classification
1.	AR (with systemic atopy)	i. Classical classification 1. Time of exposure to aeroallergen or aeroallergens: perennial, seasonal, and occupational ii. ARIA classification 1. Duration of symptoms: persistent and intermittent 2. Severity of symptoms: mild, moderate, and severe
2.	Local AR (without systemic atopy)	i. Classical classification 1. Time of exposure to aeroallergen or aeroallergens: perennial, seasonal, and occupational ii. ARIA classification 1. Duration of symptoms: persistent and intermittent 2. Severity of symptoms: mild, moderate, and severe

## 3 Pathophysiology

**Figure 2:**
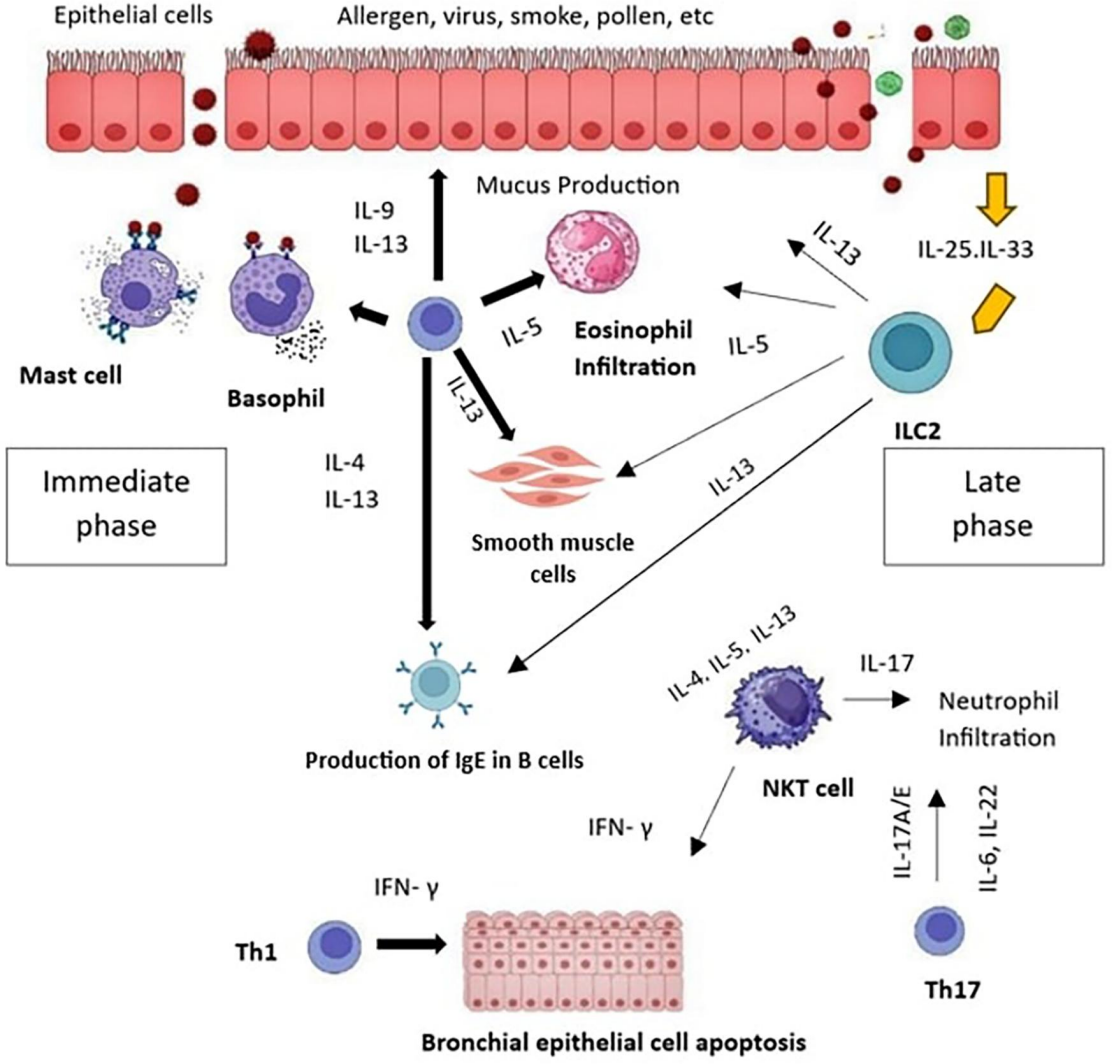
Immunologic mechanisms underlying allergic effector phases.

The clinical manifestation of AR is influenced by both the early and late phases (early and late components) of the allergic reaction in the nose. The early phase of AR is when all of its manifestations occur, and it is distinguished by the acute activation of allergy effector cells via the interaction between IgE and allergen (16). The development of nasal hyperresponsiveness and more latent symptoms, along with the inflammatory cells' recruitment and activation, are characteristics of the late phase (17). Histamine, proteases, and some cytokines, such as tumor necrosis factor (TNF), are released within five minutes of the allergen, causing deregulation of mast cells in the early phase response (the framework for a few of the acute reactions linked to AR) (18). Figure 2 illustrates immunological mechanisms that underlie the phases of allergic responses.

Recurrent symptoms (typically nasal congestion) that frequently last are caused by cytokines and mediators produced in the early stages of an immune response to an initiating allergen, which result in a second cellular inflammatory reaction that lasts for 4–8 h (late-phase inflammatory response) (19).

The late-phase response, which is characterized by nasal obstruction and hyperreactivity, is a delayed eosinophil and Th2T cell-predominant inflammation caused by newly synthesized mediators such as leukotrienes, chemokines, and cytokines (20, 21). These include interactions between the neuro-immune system and inflammatory mediators, such as the release of neurokinins and neuropeptides (calcitonin gene-related peptide, substance P) from sensory nerve endings (22).

Immunohistochemistry of nasal turbinate biopsies obtained from patients with AR either six hours after allergen challenge or during natural exposure has revealed increased expression of the lymphocyte chemokine receptors CCR3 and CCR4, eosinophil infiltration, and higher numbers of cells expressing mRNA for IL-4 and IL-5 (23–25). The inflammatory characteristics observed during late-phase reactions may be attributed to the collective mobilization of effector cells (26).

Priming is the term used to describe increased nasal responsiveness to an allergen with repeated allergen exposure. It is caused by increased mast cell counts in the epithelium, increased permeability of the epithelium, easier allergen penetration to IgE-bearing cells, and exaggerated responses of the nasal end organs (27, 28). Priming can also be caused by air pollution. The priming response may be suppressed by intranasal corticosteroid (INCS) treatment (29).

Due to underlying genetic and immunologic differences, some populations are more susceptible to allergic rhinitis. The genes TSLP, GATA3, IL-4, IL-13, and IL-33, which control Th2 cell differentiation and IgE synthesis, have polymorphisms that have been found by genome-wide association studies (30, 31). Variants in IL1RL1 and HLA-DQ have also been linked to an increased incidence of atopy and allergic airway inflammation. Those who have a family history of atopic diseases including asthma, eczema, or allergic conjunctivitis exhibit a genetically primed immune response that is marked by increased Th2 polarization and eosinophilia. Air pollution and microbiological exposure are examples of environmental stressors that can further cause epigenetic changes, such as changed DNA methylation, that affect immunological tolerance and disease manifestation (32). The development of tailored, precision-based treatments for AR and risk stratification depends on the identification of these genetic and immunologic vulnerabilities.

## 4 Diagnostic approaches

**Figure 3:**
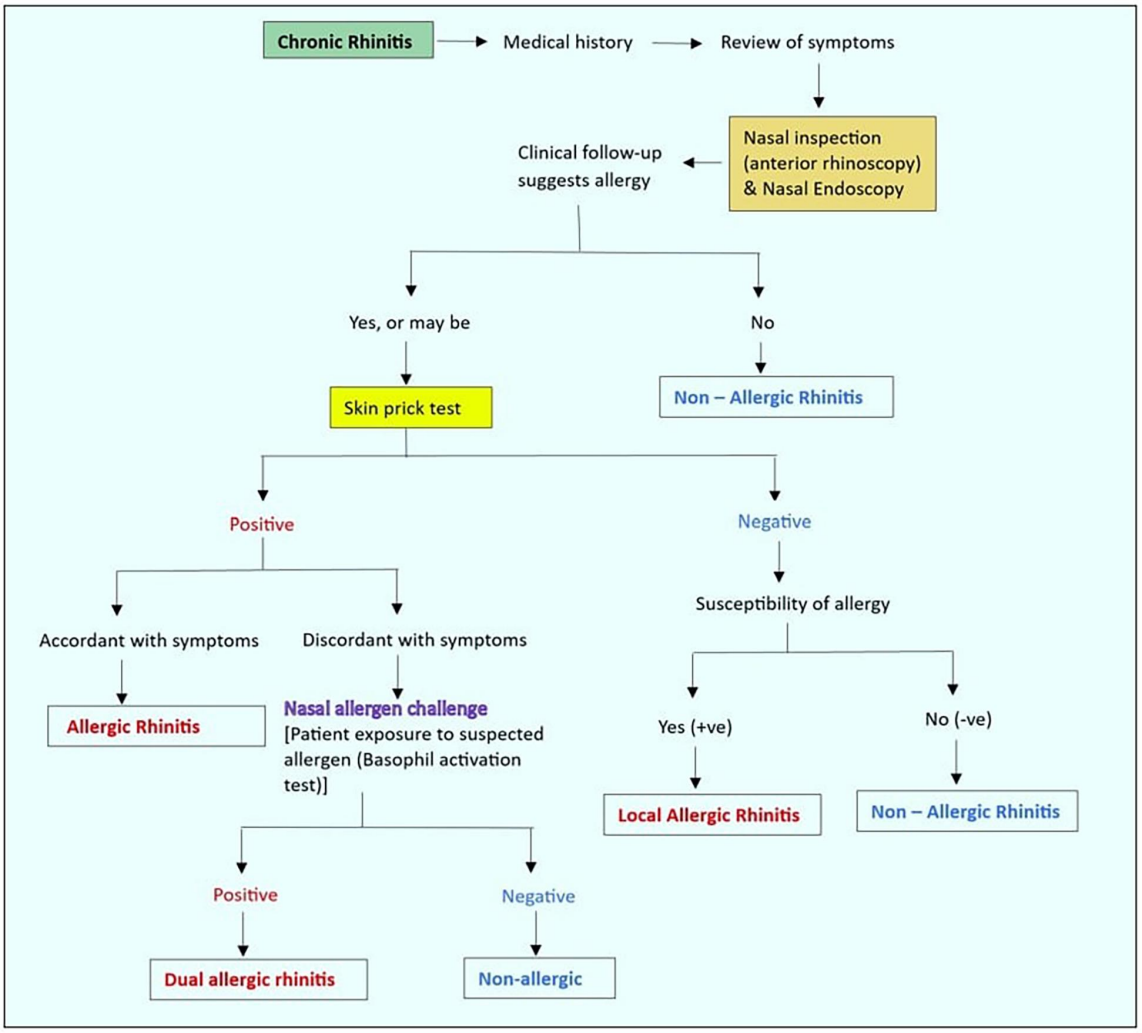
Diagnostic delineation of rhinitis.

Clear nasal secretions are frequently seen in AR, along with enlarged turbinate's and a typically pale and slack nasal mucosa (33). In order to distinguish NAR from AR and to show if IgE antibodies are present or absent, skin testing for seasonal and perennial aeroallergens is useful. If an anatomical or pathologic entity is suspected but not visible on speculum examination, rhinoscopy may be useful (34). AR is diagnosed by taking a thorough medical history, asking about potential asthma, performing a nasal examination, and, if feasible, inspecting the throat, ears, and chest. Figure 3 outlines the diagnostic delineation of rhinitis. The diagnosis is supported by specific allergy tests, such as skin prick or blood testing, for particular IgE to allergens that the history suggests (35).

For almost a century, clinical skin prick testing (SPT) has been used to help diagnose atopic, IgE-mediated disorders. It is regarded as the gold standard by which other diagnostic techniques are measured. SPT has been proven to be a reliable and effective way to identify allergies. SPT enables the assessment of several allergens in a single session, with results available in 15–20 min (36, 37).

The nasal allergen provocation test is a sensitive, specific, and reproducible method, but it takes a long time and requires expert personnel. Apart from identifying pertinent and irrelevant allergen sensitisation in atopic situations, NAPT can distinguish between allergic (AR and LAR) and non-allergic people (healthy controls and NAR) (38–40).

### 4.1 Differential diagnosis

**Figure 4:**
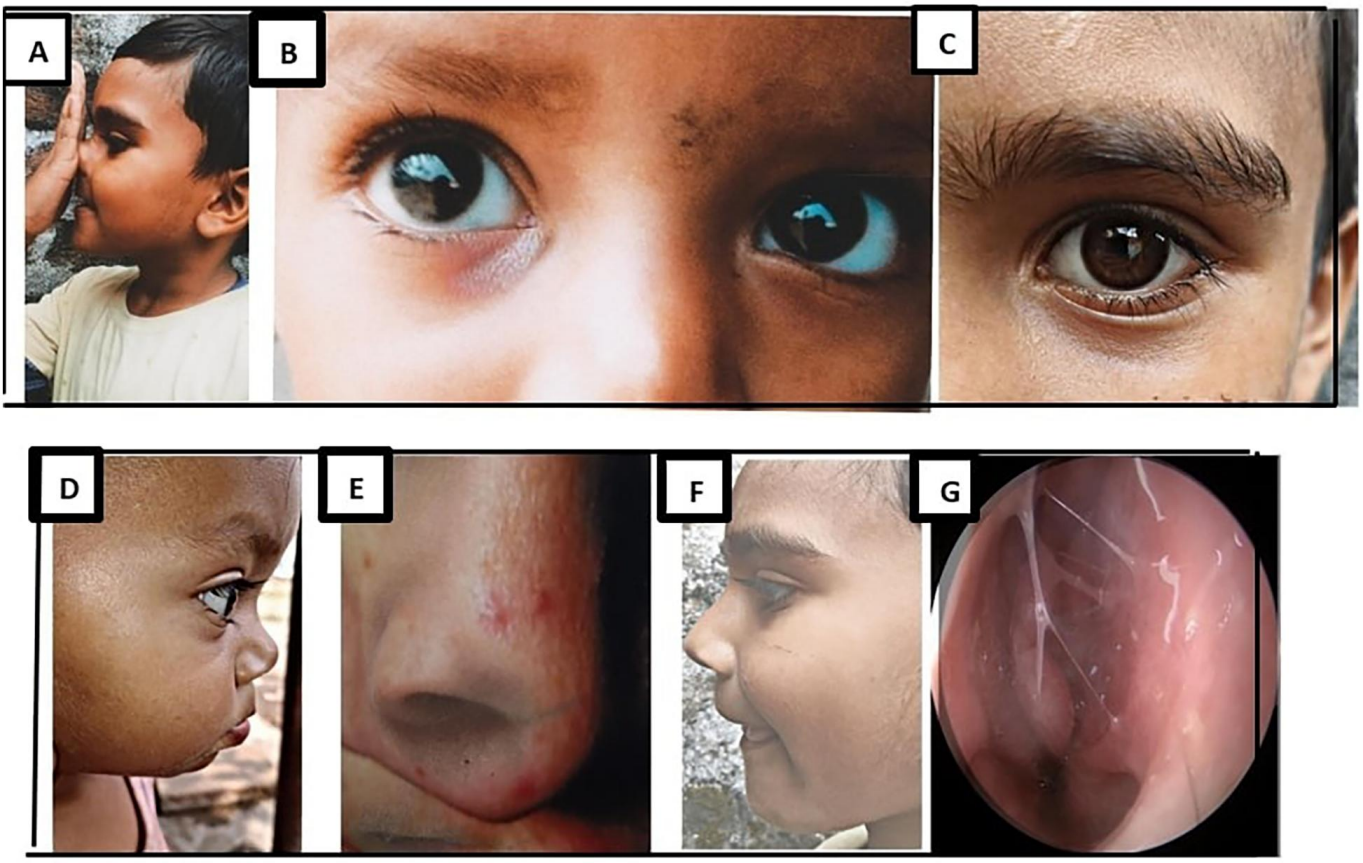
The pathophysiology of AR results in typical examination findings **(A)** the allergic salute. **(B)** Allergic shiners. **(C)** Dennie-Morgan lines. **(D)** Facial grimacing or twitching. This is related to nasal itching. **(E)** Nasal creasing related to the allergic salute. **(F)** Allergic facies. **(G)** Typical nasal mucosa.

During consultation, a few differential illnesses should be taken into account as they can resemble AR. First, inflammatory nasal pathologies such as chronic rhinosinusitis and other rhinitis causes, including vasomotor rhinitis, gestational rhinitis, and idiopathic non-AR. In addition to AR, structural causes, including congenital or traumatic septal deviation, can also cause substantial nasal obstruction. Third, nasal symptoms are more common in specialized clinics for systemic disorders such as granulomatosis with polyangiitis, sarcoid, and systemic lupus erythematosus (41). Figure 4, outlined below, depicts the typical examination findings of AR.

## 5 Treatments

**Figure 5:**
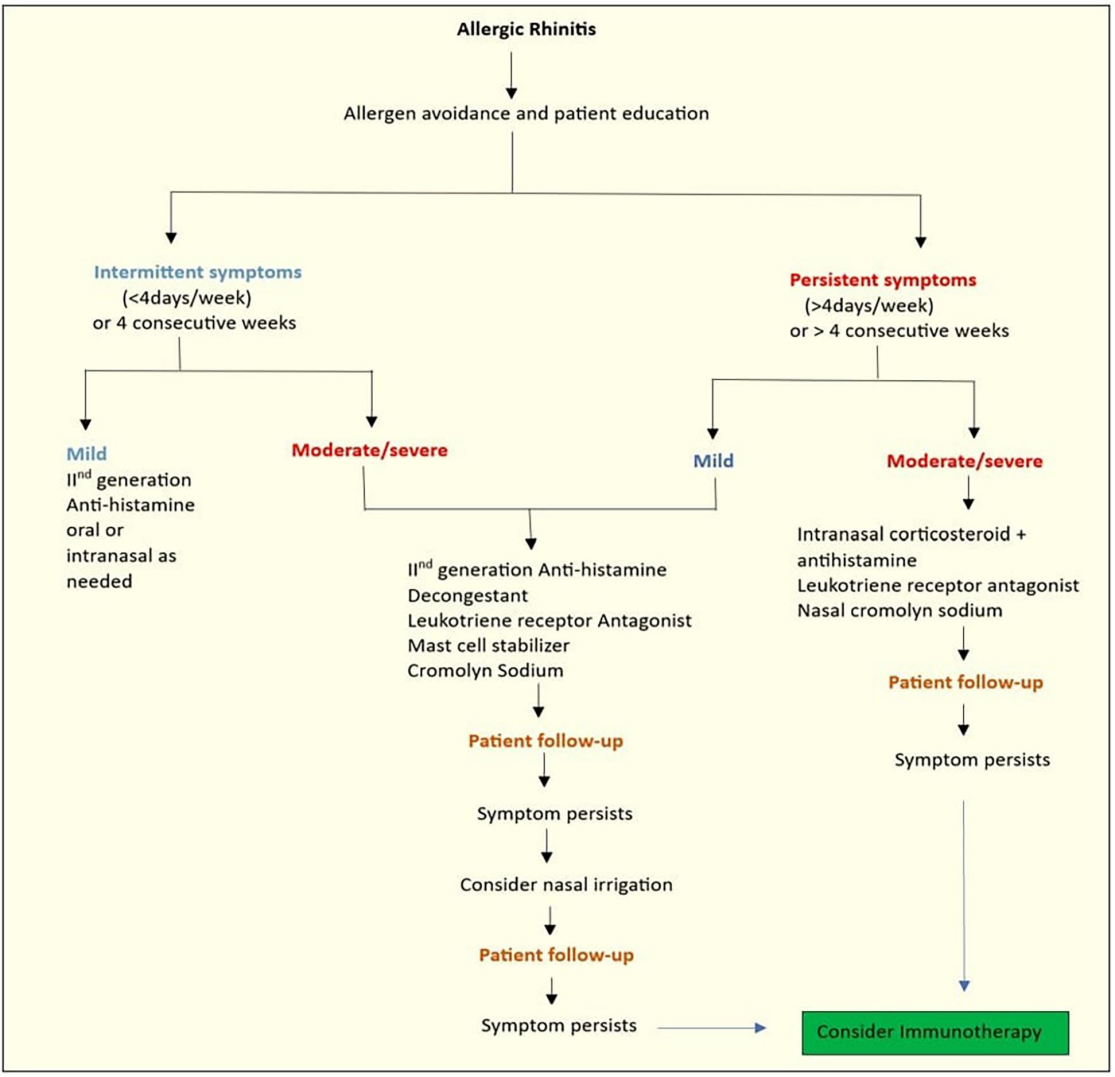
AR treatment.

Before approaching pharmacotherapy, it is essential to identify the allergic triggers of rhinitis in order to start targeted treatments that may be able to change the course of the illness (42). When choosing pharmacotherapy for patients with AR, factors such as (1) Patient empowerment, age, and preferences; (2) Prominent symptoms, symptom severity, and multimorbidity; (3) Treatment efficacy and safety; (4) Treatment onset time; (5) Current treatment; (6) Past response to treatment; (7) Impact on sleep and work productivity; (8) Self-management techniques; and (9) Resource utilization are taken into consideration (43). Figure 5 illustrates the AR treatment algorithm.

Patient education, medication, biologics, and allergen-specific immunotherapy (AIT) all belong to the management of AR (44). Mast cell stabilizers, antihistamines, glucocorticosteroids (GCSs), leukotriene receptor antagonists, and decongestants are currently the main medications used to treat AR. Allergen-specific immunotherapy (AIT) is the only causative therapeutic strategy that may alter the course of the disease (45). Avoiding allergens is an additional non-pharmacological way to lessen symptoms (46).

### 5.1 Conventional treatments

#### 5.1.1 Intranasal corticosteroids

The best drug class for managing the manifestations of AR is intranasal corticosteroids (INCSs), according to each set of practice guidelines. The capacity of INCSs to alter the pathogenesis of AR, including the recruitment and infiltration of activated inflammatory cells into the nasal mucosa and the release of many mediators and cytokines, is responsible for their great efficacy. In 2017, Bridgeman et al. Clinical data clearly showed that INCS efficiently relieves non-nasal (such as ophthalmic) symptoms as well as symptoms of AR in the early and late phases, including sneezing, nasal congestion, rhinorrhea, and nasal itching (47, 48).

The systemic bioavailability of first-generation INCSs, such as Flunisolide, Budesonide, Beclomethasone, and Triamcinolone Acetonide, is higher. Second-generation INCSs (Ciclesonide, Fluticasone Furoate, Fluticasone Propionate, and Mometasone), because of their extremely low systemic bioavailability (<1%), are less likely to cause systemic side effects (49).

#### 5.1.2 Oral antihistamine

Antihistamines (AH) that target the histamine H1 receptor are very beneficial to patients with AR in maintaining and enhancing their quality of life. Bilastine and Fexofenadine are classified as “non-brain-penetrating antihistamines” under the non-sedating group by the H1RO. Numerous chemical characteristics of these two medications are similar. Nevertheless, Bilastine binds to the H1 receptor more strongly and has a longer half-life (50).

By down-regulating the H1 receptor, H1 antihistamines function as inverse agonists to prevent histamine's pro-inflammatory effects (51). They can also produce additional unwanted deleterious effects (cardiac and other sites) by blocking transmission through ion channels and muscarinic, α-adrenergic, and serotonin receptors. Since the 1940s, oral H1 antihistamines have been available for purchase (52).

#### 5.1.3 Intranasal antihistamine

Intranasal antihistamines have been shown to have clinically relevant effects on a variety of mediators (such as histamines, leukotrienes, cytokines, chemokines, mast cells, eosinophils, and neutrophils) at clinically relevant concentrations, whereas oral antihistamines require much higher concentrations (than usual dosing) to achieve any anti-inflammatory effects. The effectiveness of intranasal antihistamines in reducing AR symptoms may be due to their local administration and distinct pharmacologic profile (53, 54).

The only AH now available in the US with indications for both AR and VMR (Vasomotor rhinitis) is intranasal Azelastine 0.1%. Only AR should be treated with Olopatadine and Azelastine 0.15%. Olopatadine and Azelastine 0.15% dose-ranging experiments have demonstrated a therapeutic onset of action within 30 min of the initial dosage and sustained efficacy over a 12-h period (55).

#### 5.1.4 Combination intranasal corticosteroid and antihistamine

For individuals with moderate to severe symptoms, combination therapy that combines an intranasal steroid with an oral or intranasal antihistamine may improve clinical outcomes. When administered together, intranasal corticosteroids and intranasal antihistamines are noticeably more effective than when administered alone (56). However, the International Consensus Statement on Allergy and Rhinology states that the INCS and oral AH combination is optional for AR and conditional for seasonal AR, according to ARIA (57).

In the study conducted by Radwan et al. compared to before treatment, nasal symptoms were considerably better in the Fluticasone and Fluticasone + Azelastine groups after one and two months (*P* value <0.001) (58).

#### 5.1.5 Oral decongestants

The over-the-counter medications known as decongestants (Oxymetazoline, Pseudoephedrine, and Phenylephrine) come in a variety of forms and combinations (59). The indication of Phenylephrine has increased since Pseudoephedrine was moved behind the counter to control access. But when it comes to treating AR, Phenylephrine has been demonstrated to be less efficient than a placebo (6).

When taken orally, Phenylephrine or Pseudoephedrine works by interacting with postsynaptic adrenergic receptors to cause the contraction of the blood vessels in the nasal mucosa (60).

But unlike oral decongestants, topical decongestants are not usually advised for long-term use because of the possibility of rebound congestion (rhinitis medicamentosa), which can happen as soon as three to five days of use, and possible damage to the nasal mucosa, including epithelial metaplasia, goblet cell hyperplasia, and mucosal edema (61).

#### 5.1.6 Intranasal cromolyns

In the conjunctiva and upper and lower airways, where mucosal mast cells play a critical role in the allergic response, it has been demonstrated that Cromolyn inhibits both the early- and late-phase allergen-induced reactions. Cromolyn lowers the release of mediators that cause inflammation and the allergic reaction by preventing sensitized mast cells from degranulating (62, 63).

Nowadays, sneezing, nasal discharge, nasal congestion, and eye discomfort are all signs of AR that are managed with cromolyn nasal sprays. Several clinical investigations have shown that cromolyn, when taken intranasally, is effective in treating AR (64–66).

#### 5.1.7 Intranasal anticholinergics

In both allergic and non-AR, topical anticholinergics, such as Ipratropium bromide nasal spray, are useful in relieving rhinorrhea symptoms (67). It has been demonstrated that the anticholinergic Ipratropium bromide, which comes in nasal form, effectively controls watery rhinorrhea by blocking the parasympathetic signaling that causes it (68).

#### 5.1.8 Leukotriene receptor antagonist

Mast cells and basophils are the primary inflammatory cells that release histamine and leukotriene (69). Monocytes and eosinophils are two more inflammatory cells that release leukotrienes. Leukotriene release causes smooth muscle contraction, mucus hypersecretion in the airways, vasodilation, and enhanced vascular permeability (70, 71). Montelukast has been demonstrated to lessen sneezing, pruritus, rhinorrhea, and congestion during the day. Montelukast is regarded as a very safe medication that can be used to treat and prevent airway allergies (72). Table 3 gives a summary of conventional treatment.

**Table 3:** Summary of conventional treatments.

S. No	Therapy/ drugs	Mechanism of action	Adverse effects	References
1.	Intranasal corticosteroids Beclomethasone Budesonide Ciclesonide Flunisolide Fluticasone furoate Fluticasone propionate Mometasone Triamcinolone acetonide	Inhibit the release of cytokines and reduce the influx of inflammatory cells	Bitter aftertaste, burning, epistaxis, headache, nasal dryness; possible systemic absorption, rhinitis medicamentosa, stinging, throat irritation	Sur et al. (2015) (73)
2.	Oral antihistamine Cetrizine Loratadine Fexofenadine Desloratadine Levocetrizine	Blocks Histamine H_1_ receptors	Dry mouth, drowsiness, fatigue	Lehman et al. (2006) (74)
3.	Oral decongestants Pseudoephedrin Phenylephrine	Alpha adrenergic agonist that cause nasal vasoconstriction	Palpitations, irritability, nasal dryness, hypertension, tremor, sleep disturbance, loss of appetite, urinary retention, dizziness, tachycardia	Platt et al. (2014) (75)
4.	Intranasal cromolyns Cromolyn	Mast cell stabilizer: inhibit Histamine release	Bad taste, cough, throat irritation	Sarbacker et al. (2016) (76)
5.	Intranasal anticholinergics Ipratopium	Acetylcholine receptor blocker	Nasal dryness, headache	Tahir et al. (2020) (77)
6.	Leukotriene receptor antagonist Monteleukast	Leukotriene receptor blocker	LTRAs are well tolerated with few reported negative results, e.g., sneezing/fatigue, headache (5%), angioedema, pulmonary eosinophilia, rashes, dry mouth, upper respiratory tract infection, abdominal pain, dizziness, pruritus, sinusitis, arthralgia, and nasopharyngitis	Alzughaibi et al. (2021) (78)

## 6 Overview of AR conventional treatment

### 6.1 Allergen immunotherapy

The sole disease-modifying treatment available at the moment, allergen immunotherapy (AIT), is chosen by a percentage of patients who do not respond to traditional medication. Both the sublingual (SLIT) and subcutaneous (SCIT) routes can be used to give AIT. Both administration methods are secure, efficient, and can produce tolerance that lasts for years after stopping therapy (79). For example, SLIT uses allergen extracts administered orally to produce therapeutic effects and mucosal immune modulation. By subcutaneous injection, SCIT, on the other hand, progressively improves the patient's tolerance to particular allergens, reducing inflammatory and allergic reactions. Both Immunotherapies have demonstrated some potential for treating asthma and AR (80). Although AIT continues to be the only treatment for allergic disorders with the potential to be curative, it still has several issues with patient adherence, efficacy, security, and long duration (81).

## 7 Phytotherapy for the management of AR

The quest for herbal medications to treat AR is in progress, despite the fact that the use of phytotherapy is widely viewed with scepticism and criticism (82). Among the TCIM fields (traditional, complementary, and integrative medicine), phytotherapy (PT) is frequently utilized by people with SAR and may be a helpful therapeutic choice. PT uses plant preparations, including exudates like gum, and plant components like seeds, leaves, and bark to treat or prevent illnesses (83). Given the extensive and expanding usage of herbal therapy as well as their possible pharmacodynamic and pharmacokinetic effects, doctors would benefit from knowing how HTs (herbal therapies) are used for different illnesses (84). A few herbal therapies for AR are summarized in Table 4 below.

**Table 4:** Phytotherapy intervention for AR.

S.no.	Plant	Phytochemicals (Class)	Pharmacological Profile	Model/Mechanism	Clinical Evidence
1.	*Sinomenium acutum*	sinomenine (alkaloid)	Alleviate the symptoms of AR in mice and has an immunosuppressive effect on AR Chen et al. (2017) (85)	*In vivo* (mice); suppresses T-cells	No clinical studies identified for AR. Used traditionally in Chinese medicine.
2.	*Astragalus membranaceus*	Astragalus polysaccharides (Polysaccharide)	alleviates the nasal symptoms and histopathological changes of eosinophil infiltration, goblet cell metaplasia, and collagen deposition in AR rats and inhibited OVA-sIgE, histamine, and Th2-related cytokine secretion Xu et al. (2021) (86)	*In vivo* (rats)	Double-blind RCT (6 weeks) in SAR patients confirming efficacy & safety Matkovic et al. (2010) (140)
3.	*Curcuma longa*	Curcumin (Polyphenol)	Inhibit mast cell activation Kinney et al. (2015) (87)	*in vivo* & *in vitro*	Pilot RCTs show symptom relief, improved airflow, reduced pro-inflammatory markers Wu et al. (2016) (99)
4.	*Urtica dioica (Stinging Nettle*	Flavonoids, histamine & prostaglandin inhibitors (Flavonoid)	Inhibits H₁ receptors, reduces eosinophilic inflammation	*in vitro*, *in vivo*, clinical	RCTs (1990, 2017) show symptom relief, eosinophil reduction Bakhshaee et al. (2017) (100)
5.	*Piper nigrum*	Piperine (Alkaloid)	Inhibit eosinophil activation, inhibit early phase nasal symptoms, block infiltration of inflammatory cells Kim et al. (2009) (88)	*in vivo* (guinea pigs)	No clinical data
6.	*Scutellaria baicalensis*	Baicalin (Flavonoid)	Inhibit release of histamine, beta-hex expression Zhou et al. (2016) (89), Chen et al. (2019) (90)	*in vitro* (RBL cells)	No clinical study
7.	*Tussilago farfara L.*	Sesquiterpene (Terpenoid)	Improve rhinitis is symptoms, alleviate pathological changes of nasal mucosa, decrease the production of IgE, histamine and IL-6 in ova albumin induced AR in guinea pigs Jin et al. (2020) (91)	*in vivo* (guinea pigs)	No human trial
8.	*Petasites hybridus (Butterbur)*	Petasins (Sesquiterpene ester)	Blocks leukotriene/prostaglandin production & mast-cell degranulation	*In vitro*, *in vivo*, clinical	Multiple RCTs vs placebo and antihistamines; strong efficacy, low AEs Kaufeler et al. (2006) (101)
9.	*Perilla frutescens*	Flavanone (Flavonoid)	Inhibit IgE mediated histamine release, supress passive cutaneous anaphylaxis, prevent nasal symptoms in murine model Kamei et al. (2017) (92)	*In vivo* (mice)	RCT (2004) showed reduced allergic rhinoconjunctivitis symptoms; nutraceutical studies support benefit Takano et al. (2004) (102)
10.	*Dictamenine dasycarpus Turcz*	Furoquinoline alkaloid (Alkaloid)	Inhibit the molecular signaling pathway mediated by LYN kinase during the activation of mast cell Liu et al. (2023) (93), Zheng et al. (2018) (94)	*In vitro* (cell signaling)	No clinical evidence
11.	*Cissampelos sympodialis*	Warifteine (Alkaloid)	Shown to reduce immediate allergic reactions and thermal hyperalgesic response in sensitized animal Vieira et al. (2018) (95)	*In vivo* (mice)	No human data
12.	*Perilla frutescens*	Luteolin (Flavonoid)	Supress IgE, IgG, IL-4 in AR induced in mice model Jeon et al. (2014) (96), Liang et al. (2020) (97), Dong et al. (2021) (98)	*In vivo* (mice)	No clinical study

## 8 Innovative strategies under investigation/development for AR

This chart describes various new approaches to treating allergic rhinitis, such as acupuncture, advanced pharmacology (e.g., MP29-02, BLU-808), probiotics (LP-33, LH2171), bioabsorbable sinus implants etc. These therapies are supported by recent research and cover a range of delivery modalities, including oral, intranasal, and implant-based techniques. Table 5 depicts novel treatments under development.

**Table 5:** Novel treatment under development.

S.no.	Interventions	Tools/drugs	Description	Administration route	References
1.	Patients’ education	MASK-allergy diary	–	Smartphone/Internet	Meng et al. (2019) (103)
2.	Pharmacotherapy	ABH MP29-02 BLU-808	Arginase inhibitor 2(S)-amino-6-boronohexanoic acid Combination nasal spray of azelastine hydrochloride and fluticasone propionate Oral wild type KIT inhibitor	Inhalational Intranasal Oral	Meng et al. (2019) (103) Meng et al. (2019) (103). Grassian et al. (2024) (104)
3.	*Probiotics*	LP-33 LH2171	Lactobacillus paracasei Lactobacillus helveticus	Oral	Liu et al. (2022) (105)
4.	*Bioabsorbable sinus implants*	Propel,Sinuva	Mometasone furoate	Sinus implants	Patel et al. (2020) (141)
5.	*Acupuncture*	Sphenopalatine ganglion acupuncture	–	Acupuncture	Wang et al. (2016) (106)
6.	*Intralymphatic Immunotherapy*	Intra-lymph node injections of modular allergen translocation (MAT)-Fel d 1 vaccine	Encourage the development of allergen-specific peripheral T-cell tolerance, inflammasome activation, and cellular internalization of the allergen.	Intra-lymph node injections	Zhang et al. (2022) (107)
7.	*Intradermal immunotherapy (IDIT) and epicutaneous immunotherapy (EPIT)*	Administering the allergen through the skin	Many immune cells, notably dendritic cells, in the dermis can process and deliver allergens to T-cells, boosting immunological tolerance.	Subcutaneous injections	Sola et al. (2020) (108)
8.	Monoclonal antibody	*LP-003 CM310 (Spectrobab) Anti-IL-33 (Etokimab)*	Suppress the expression of the reporter gene and prevent human IgE from attaching to Fc*ε*RI and FcεRII (CD23) receptors. Target the human interleukin-4 receptor alpha subunit (IL-4R α) Block early Th2 signaling, reducing inflammation and symptom severity	Subcutaneous injections subcutaneous injection subcutaneous injection	Guan et al. (2025) (109). Cheng et al. (2025) (110). Obata-Ninomiya et al. (2024) (111)

## 9 Market evolution

The United States alone has 8–20 million individuals receiving medication, and the annual global sales of medications for AR have surpassed $6 billion. Given that AR is equally prevalent in both established and emerging economies as other major chronic illnesses, such as diabetes, high cholesterol, and hypertension, the market potential is significant (112). Table 6 summarizes the accessible commercial formulation for the pharmacotherapy of AR.

**Table 6:** Commercial formulations.

S.no.	Drug	BCS class	Formulation	Proprietary name	Manufacturing corporation	References
1.	*Ciclesonide*	II	Nasal spray	Omnaris	Nycomed	Meltzer et al. (2014) (113)
2.	*Montelukast*	II	Oral tablet	Singulair	Merck & Co.	Young et al. (2012) (114)
3.	*Ipratropium*	III	Nasal spray	Atrovent	Boehringer Ingelheim	Pfister et al. (1996) (115)
4.	*Cromolyn*	III	Nasal spray	Nasalcrom	Emerson healthcare	Strauss et al. (2020) (63)
5.	*Azelastine*	IV	Nasal spray	Astelin	Meda pharmaceuticals Inc.	Horbal et al. (2010) (116)
6.	*Olopatadine*	III	Nasal spray	Patanase	Alcon	Roland et al. (2010) (117)
7.	*Loratadine*	II	Oral tablet	Claritin	Merck & Co.	Kay et al. (1999) (118)
8.	*Levocetrizine*	III	Oral tablet	Xyzal	Sanofiaventis	Hair et al. (2006) (119)
9.	*Fexofenadine*	III	Oral tablet	Allegra	Sanofi India Ltd.	Tokumura et al. (2016) (120)
10.	*Beclomethasone*	II	Nasal spray	Beconase	Omega pharma Ltd.	Beconase et al. (2019) (121)
11.	*Budesonide*	II	Nasal spray	Rhinocort	Astrazeneca pharma india Ltd.	Creticos et al. (1998) (122)
12.	*Phenylephrine*	III	Oral tablet	Sudafed PE	Johnson and johnson	Khoshnevis al. (2023) (123)
13.	*Fluticasone furoate*	II	Nasal spray	Veramyst	Glaxosmithkline consumer healthcare	Ahsanuddin et al. (2021) (124)
14.	*Fluticasone propionate*	II	Nasal spray	Flonase	Glaxosmithkline consumer healthcare	Messina et al. (2019) (125)
15.	*Mometasone*	II	Nasal spray	Nasonex	Merck & Co.	Minshall et al. (1998) (126)
16.	*Triamcinolone*	IV	Nasal spray	Nasacort	Sanofi	Hochhaus et al. (2002) (127)
17.	*Bilastine*	II	Oral tablet	Blexten	Aralez pharmaceuticals Canada Inc.	Tim et al. (2020) (128)

## 10 Ongoing clinical trials for potential AR pharmacotherapy

Even with the availability of proven therapy approaches, a sizable percentage of patients remain uncontrolled (129). Additionally, even though the majority of corticosteroid nasal sprays have a high safety profile, linked side effects to treatments for AR, such as sleepiness and disorientation, bitter taste, epistaxis, headache, somnolence, and nasal burning, have prompted increased efforts to find new medications (Consensus report 1994) (130). Here, we have summarized potential pharmacotherapy for the treatment of AR that could emerge from advancing clinical trials. Supplementary Table 7 illustrates the ongoing clinical trials for the treatment of AR.

## 11 Public health implications

Environmental and lifestyle variables related to industrialization and rising westernization are unquestionably linked to the growth in allergy disorders (131). Law et al. estimated that the direct medical costs of AR were 3.4 USD billion, of which 46.6% could be attributed to prescription medicine, using data from the 1996 Medical Expenditure Panel (132, 133).

Being a chronic condition, AR has substantial direct expenses related to managing the illness, indirect costs related to decreased productivity at work, and “hidden” costs associated with managing comorbidities that are typical of AR patients (134). Furthermore, AR is a systemic inflammatory illness that frequently coexists with other conditions such as otitis media, sinusitis, conjunctivitis, asthma, and atopic disease, making it more difficult to manage and treat AR patients (135).

Common inflammatory pathways and immunological mediators including IL-4, IL-5, and IL-13 cause allergic rhinitis (AR) to coexist with asthma, conjunctivitis, and atopic dermatitis. Clinical research supports the idea that “one airway, one disease” by showing that almost half of AR patients acquire asthma and that the majority of asthmatics have symptoms of rhinitis (133). According to epidemiological research, up to 40%–50% of patients with AR go on to acquire asthma, while over 70% of those with asthma also have symptoms of rhinitis, highlighting the reciprocal association between these conditions (134). Recent mechanistic research has shown that increased expression of IL-1β and inducible nitric oxide synthase (iNOS) in perennial AR can lead to bronchial inflammation and decreased lung function, indicating that AR may actively encourage the development of asthma rather than just coexist with it (135). This overlap highlights the necessity of coordinated care of allergic illnesses by indicating that AR may function as a marker and a cause of lower airway inflammation.

The illness has, however, been underappreciated up to this point since people with milder symptoms are less likely to seek medical attention and see a doctor; as a result, they are frequently underdiagnosed and hence result in undertreatment (136). For patients and medical personnel, the best possible management of AR is still crucial despite the pandemic and the constantly changing post-pandemic circumstances (137).

Qualitative results emphasized the value of healthcare professional assistance and patient education in controlling AR (138). Identifying the most hazardous particulate matter and its constituents, lowering environmental exposures, focusing on control laws, improving protection for vulnerable groups, and lessening the financial and health burden on public health are all desirable (139).

## 12 Conclusion

In summary, AR is a common illness that has a major negative influence on many people's quality of life. It is brought on by the immune system's reaction to allergens and manifests as symptoms including itching, sneezing, and congestion in the nose. Despite the fact that its precise pathophysiology entails intricate relationships between genetic predisposition and environmental circumstances, there are effective management techniques accessible, such as avoiding allergens and using pharmaceutical therapies.

Future research is increasingly focused on more targeted and durable treatment strategies. Novel strategies like gene therapy have the potential to alter the underlying immune response in genetically predisposed people. Furthermore, environmental interventions could be crucial in lowering the burden of disease. These could include anything from smart home technologies that monitor and limit exposure to precise allergen mapping. The next generation of AR management is probably going to be shaped by personalized medicine, which incorporates environmental risk assessment and genomic data. To improve long-term results and the quality of life for those who suffer from allergic rhinitis, these areas must be advanced.
