# Supplementary material for: Unravelling allergic rhinitis: exploring pathophysiology, advances in treatment, and future directions
Source: Front Allergy. 2025 Dec 4;6:1636415. doi: 10.3389/falgy.2025.1636415 (PMC12711840; doi:10.3389/falgy.2025.1636415)
Supplement: Supplementary file 1 [file Table1.docx]

**Supplementary Material**

**Table 7- Clinical trials for treatment of AR [122]**

| S.no. | Project title | NCT number | Eligible age for participation | Intervention | Concurrent trial phase status | Funding entity |
| --- | --- | --- | --- | --- | --- | --- |
| 1. | Mechanism of EGR2 in AR | NCT05724290 | 16 Years to 76 Years (Child,  Adult,  Older Adult ) | Genetic: EGR2 | Phase II | Zu-xia Ma |
| 2. | Study of Yupingfeng Powder Treating AR (AR) (RCMARb) | NCT06608017 | 18 Years and older (Adult,  Older Adult ) | Drug: Chinese Herbal Medicine granules | Phase II Phase III | Chinese University of Hong Kong |
| 3. | Clinical Study of AR Therapy by Stem Cell | NCT05151133 | 18 Years to 60 Years (Adult) | Biological: Low dose UCMSCs  Biological: Moderatedose UCMSCs  Biological: High dose UCMSCs | Phase I | Yantai Yuhuangding Hospital |
| 4. | A Phase II Study Evaluating Intranasal GSK256066 and Azelastine Hydrochloride in Subjects With Seasonal AR | NCT00612118 | 18 Years to 50 Years (Adult) | Drug: GSK256066  Drug: azelastine hydrochloride | Phase II | GlaxoSmithKline |
| 5. | Safety and Efficacy Study of PA9159 Nasal Spray for the Treatment of Seasonal AR | NCT05234580 | 12 Years and older (Child,  Adult,  Older Adult) | Drug: PA9159 nasal spray solution | Phase II | Anhui Palo Alto Pharmaceuticals, Inc. |
| 6. | Study of CM310 in Patients With AR | NCT05908721 | 18 Years to 65 Years (Adult,  Older Adult ) | Biological: CM310 | Phase II | Keymed Biosciences Co.Ltd |
| 7. | A Study of IL4Rα Monoclonal Antibody in Patients With Uncontrolled Seasonal AR. | NCT06028490 | 18 Years to 75 Years (Adult,  Older Adult ) | Biological: GR1802 | Phase II | Zheng Liu ENT |
| 8. | Efficacy and Safety of MAZ-101 in the Treatment of Persistent AR (PER) | NCT05684380 | 12 Years and older (Child,  Adult,  Older Adult ) | Drug: MAZ-101 association  Drug: DYMISTA® | Phase III | EMS |
| 9. | [Efficacy and Safety of DEP114 in the Treatment of Moderate to Severe Persistent AR in Children.](https://clinicaltrials.gov/study/NCT06272409?cond=Allergic%20Rhinitis&aggFilters=phase:1%202%203%200&page=3&rank=26) | NCT06272409 | 6 Years to 11 Years (Child ) | Drug: DEP114  Drug: Desloratadine 0.5 MG/ML | Phase III | EMS |
| 10. | A Study Assessing the Safety and Effectiveness of FEX60/​PE10 Fixed Combination Tablet in Patients With AR | NCT02401191 | 15 Years and older (Child,  Adult,  Older Adult ) | Drug: FEX60/PE10 | Phase III | Sanofi |
| 11. | Immunological Mechanisms of Oralair® in Patients With Seasonal AR | NCT02014623 | 16 Years to 65 Years (Child,  Adult,  Older Adult ) | Other: Grass pollen sublingual immunotherapy tablet  Drug: Control | PhaseIV | Bayside Health |
| 12. | The Efficacy and Safety of Sanfujiu on Patients With Persistent AR: Randomized Controlled Study | NCT02192645 | 18 Years and older (Adult,   Older Adult ) | Drug: Sanfujiu | Phase III | Guangdong Provincial Hospital of Traditional Chinese Medicine |
| 13. | Safety of Lymph Node Injection for Allergen Immunotherapy | NCT01982474 | 15 Years to 24 Years (Child,  Adult ) | Biological: Grass pollen extract  Other: Placebo injection | Phase I | Amber Patterson |
| 14. | Evaluation of Efficacy and Safety of Specific Immunotherapy With Modified Allergen Extracts of House Dust Mites | NCT01013116 | 18 Years to 60 Years (Adult ) | Biological: modified allergen extract of house dust mites  Biological: placebo | Phase III | Roxall Medizin |
| 15. | Efficacy/​ Safety of Product CM9241GRU in Patients With Perennial AR (RESPIRE) | NCT06577077 | 18 Years and older (Adult,   Older Adult ) | Drug: Product CM9241GRU  Drug: Placebo | Phase III | Ache Laboratorios Farmaceuticos S.A. |
